# Hyperbaric oxygen therapy and cancer—a review

**DOI:** 10.1007/s11523-012-0233-x

**Published:** 2012-10-02

**Authors:** Ingrid Moen, Linda E. B. Stuhr

**Affiliations:** Department of Biomedicine, University of Bergen, Jonas Lies vei 91, 5009 Bergen, Norway

**Keywords:** Hyperbaric oxygen therapy, Cancer, Hypoxia

## Abstract

Hypoxia is a critical hallmark of solid tumors and involves enhanced cell survival, angiogenesis, glycolytic metabolism, and metastasis. Hyperbaric oxygen (HBO) treatment has for centuries been used to improve or cure disorders involving hypoxia and ischemia, by enhancing the amount of dissolved oxygen in the plasma and thereby increasing O_2_ delivery to the tissue. Studies on HBO and cancer have up to recently focused on whether enhanced oxygen acts as a cancer promoter or not. As oxygen is believed to be required for all the major processes of wound healing, one feared that the effects of HBO would be applicable to cancer tissue as well and promote cancer growth. Furthermore, one also feared that exposing patients who had been treated for cancer, to HBO, would lead to recurrence. Nevertheless, two systematic reviews on HBO and cancer have concluded that the use of HBO in patients with malignancies is considered safe. To supplement the previous reviews, we have summarized the work performed on HBO and cancer in the period 2004–2012. Based on the present as well as previous reviews, there is no evidence indicating that HBO neither acts as a stimulator of tumor growth nor as an enhancer of recurrence. On the other hand, there is evidence that implies that HBO might have tumor-inhibitory effects in certain cancer subtypes, and we thus strongly believe that we need to expand our knowledge on the effect and the mechanisms behind tumor oxygenation.

## Search terms

Pubmed was searched for articles concerning hyperbaric oxygen (HBO) and cancer for the period from 2004 to 2012, using the MeSH search terms (“hyperbaric oxygenation” and/or “hyperoxia” and “neoplasms”). A total of 28 articles were found relevant, directly involving the use of HBO as a stand-alone or as adjuvant treatment on different cancer types. We focused on growth, cell survival, angiogenesis, and metastasis observed in HBO-treated cancers the last 9 years, both as stand-alone and adjuvant treatment, and compared them to older publications involving the selected topic.

## Background

### Cancer and hypoxia

Solid tumors often contain areas subjected to acute or chronic hypoxia [1], though with variable severity in patients both within and among different tumor types [2]. Although severe or prolonged hypoxia is deleterious, adaptation to the hypoxic microenvironment has allowed cancer cells to survive and proliferate in this hostile milieu [3]. Tumor hypoxia develops due to the structural and functional abnormalities of the tumor vasculature since cancer growth often overrides the ability of the cancer vasculature to adapt to the increasing oxygen demand.

**Fig. 1:**
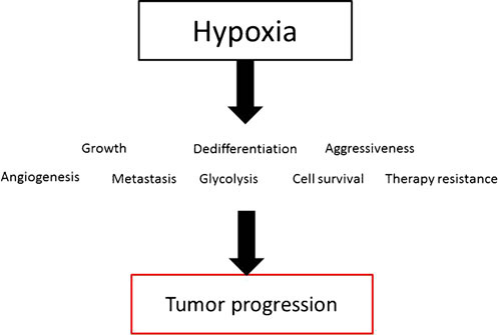
Hypoxia is a hallmark of solid tumors. Summary of the hypoxia-induced factors influencing cancer growth and progression

Traditionally, hypoxia was thought of as a factor limiting cancer growth by reducing the ability of cells to divide. However, more recently, hypoxia has proven to be a causative factor in many pathophysiological events, including cancer progression. Multiple reports have demonstrated that decreased oxygen tension selects for more malignant cells and induces multiple cellular adaptations, which again sustains and fosters cancer progression and thereby induces cancer growth (Fig. 1). Hypoxia is reported to result in cellular responses which improve oxygenation and viability through induction of angiogenesis, an alteration in metabolism by increased glycolysis and upregulation of genes involved in cell survival/apoptosis [4]. Hypoxia has also been shown to increase genetic instability, activate invasive growth, and preserve the undifferentiated cell state [1, 3]. Studies have demonstrated that hypoxia is implicated in the resistance to conventional therapy [5]. Oxygen concentration has an especially crucial role in radiation oncology and radiation resistance [6, 7]. The epithelial-to-mesenchymal transition in cancer has been shown to be induced by hypoxic conditions [8], leading to cancers with an invasive or metastatic phenotype [9]. Given its important role as a negative prognostics and predictive factor, hypoxia is considered as one of the best targets in cancer treatment.

The dual role of oxygen leads to the question: will lack of oxygen inhibit cancer progression, or is hyperoxygenating the tumor tissue the way to go in order to prevent cancer growth and development?

### Hyperbaric oxygen

Hyperbaric oxygen can be used to overcome hypoxia. HBO is based on administration of 100 % oxygen at higher than normal atmospheric pressure. HBO treatment enhances the amount of dissolved oxygen in the plasma, thereby increasing O_2_ tissue delivery independent of hemoglobin [10]. As in normal tissue, the pO_2_ in cancer tissue increases significantly during HBO exposure [11]. Thus, elevation of the tumor oxygen pressure has been shown to be preserved clinically for approximately 30 min after HBO exposure [12, 13]. HBO therapy is today accepted and routinely used for many disorders, related to both ischemia and/or hypoxia [10]. HBO is considered safe and complications are rare using today’s standard treatment protocols. The Undersea and Hyperbaric Medical Society has a list of approved indications for HBO therapy, including decompression sickness, severe carbon monoxide poisoning, nonhealing wounds, and late radiation injury.

As oxygen is believed to be required for all the major processes involved in wound healing, including resistance to infection, activation of fibroblasts, collagen deposition, angiogenesis, and epithelization [14], it has been feared that HBO would have a proliferative effect in cancers. Thus, for many decades, the focus has been to elucidate if HBO promotes cancer growth. In the early 2000s, both Feldmeier et al. [15] and Daruwalla et al. [16] reviewed the literature concerning HBO and cancer. The reviews included both experimental and clinical studies using different types of cancers, with and without additional therapy, and the results showed varied responses. Nevertheless, the conclusion in both reviews was that HBO did not promote cancer growth, and that the use of HBO in patients with malignancies was considered safe.

There are extensive studies on the effect of HBO on normal tissue and wounds. Interestingly, evidence implies that cancer tissue might differ in response from normal tissue. The studies performed on HBO and cancer are complex due to a wide range of experimental designs and treatment regimes. Nevertheless, in an attempt to clarify the differences in response to oxygenation, we have summarized the literature concerning the effect of HBO on crucial hallmarks of cancer, the effect of HBO on chemo- and radiation therapy, and in addition we have clustered the different cancer type responses.

### HBO and cell survival

Studies of prolonged hyperoxia have shown that elevated levels of reactive oxygen species (ROS) overwhelm the antioxidant defense and lead to cellular damage and possible organ dysfunction [17]. The tissue damage is found to be dependent on the cell type, concentration of oxygen, and the duration of the exposure. Gore et al. [17] have summarized the molecular mechanisms behind hyperoxia-induced cell death, revealing a complex signaling system including protein kinases and receptors such as RAGE, CXCR2, TLR3, and TLR4.

Studies of apoptosis in neoplasms treated with HBO are limited. Two in vitro studies on mammary and oral cancer cells, respectively, showed no change in apoptosis after HBO [18, 19]. On the other hand, Chen et al. [20] observed activation of the pro-apoptotic pathway MAPK and downregulation of the anti-apoptotic ERK pathway in hematopoetic cells after HBO. Additionally, a study of HBO using osteosarcoma cells also demonstrated induction of apoptosis [21]. In two different animal models, gliomas and mammary tumors, respectively, our group has demonstrated induction of cell death after HBO treatment [22–24].

Furthermore, reduced cell proliferation, together with a significant change in histology, has also been shown after HBO treatment in DMBA-induced mammary tumors in vivo [22, 24]. Granowitz et al. [18] observed the same reduction in cell proliferation in their mammary in vitro study. In addition, two recent studies on osteosarcoma cells [21] and nasopharyngeal carcinoma [25] support inhibition of cell division after HBO treatment.

Together, this might imply that changes in oxygen concentration influence antioxidant pathways [26], leading to a change in cell survival signaling. However, the picture is complex, and mechanistic studies are required before any final conclusions can be drawn.

### HBO and angiogenesis

Today, angiogenesis is proposed to be a key factor for cancer growth and metastasis. Thus, large experimental studies and clinical trials have investigated the effect of antiangiogenic therapies in the treatment of cancers. Since HBO in general has been shown to promote cellular and vascular proliferation in normal tissue and wounds (although the mechanisms are not fully understood), it was assumed that it would also induce angiogenesis in cancers. In contrary to what is expected and addressed in the literature, HBO has been shown to induce an antiangiogenic effect in two mammary tumor models [22, 24, 37], in addition to one glioma model [23]. Furthermore, multiple studies showed no change in angiogenesis after HBO treatment [27–32]. In his review, Feldmeier et al. [15] thoroughly discussed oxygen and tumor angiogenesis, underlining the difference between cancer tissue and wounds and concluded that HBO is not likely to enhance tumor angiogenesis. Thom [31] commented on the fact that the influence HBO has on hypoxia-induced factor isoform expression appears to vary with different tissues and possibly with chronology (e.g., looking early or late after wounding or an ischemic insult). We believe it to be important to distinguish between normal or injured tissue and tumor tissue when it comes to the effect of HBO and angiogenesis since there is no evidence for enhanced angiogenesis in cancerous tissue.

### HBO and metastasis

In 1966, Johnson and Lauchlan first raised concern that HBO might have metastatic potential [33]. However, it was not possible to show a statistically significant increase in the number of patients with distant metastasis, as the number of patients in the series was too small. Nevertheless, special attention was given to metastatic growth because the first reports suggested that HBO might be affecting this part of tumor progression [34]. Metastasis is a complex process requiring multiple steps, including local tumor cell invasion, entry into the blood or lymph vessels, and re-penetration and colonization at a distant site [35]. Eventually, angiogenesis is also required for distant metastasis to form.

So far, only observational studies have been performed, and studies of the effect of HBO on the individual steps of the metastatic process are still lacking [34]. None of the studies reviewed showed induced metastasis after HBO [21, 36–39]. Furthermore, a recent study found HBO to induce a mesenchymal-to-epithelial transition (MET) in DMBA-induced mammary tumors, leading to a less aggressive tumor type [24], thus indicating that oxygen might be a key factor in MET [40]. This transition should lead to cancers with a less invasive and metastatic phenotype.

### HBO and chemotherapy

Hypoxia has been described as an important factor for chemotherapeutic resistance [5]. Teicher [41] underlined that the importance of hypoxia on the response to chemotherapy is highly drug dependent. However, hypoxia-mediated chemoresistance has been ascribed to: (1) altered cellular metabolism reducing drug cytotoxicity; (2) the redox state, meaning that oxygen is required to generate ROS to be maximally cytotoxic; and (3) genetic instability, which can lead to more rapid development of drug-resistant cells. In addition to the cytotoxicity, availability of the chemotherapeutic drug in high enough dose is important to obtain a maximal effect. Tumor tissue anatomy influences transport of intravenously injected substances to the cancer cells, and thus determines the efficacy of the drug.

Al-Waili et al. [42] summarized the potential role of HBO in combination with conventional therapies. They hypothesized that HBO could improve and help overcome chemotherapeutic resistance by increasing both tumor perfusion and cellular sensitivity [42].

Studies on HBO as a chemotherapeutic adjuvant have shown augmented effects both in vitro [18, 21, 25, 43] and in vivo [21, 44–47], although the mechanism(s) are not known. Heys et al. [28] studied the effect of HBO on chemotherapy in a clinical setting, using HBO as a pretreatment to improve vascularity, and thereby improve the effect of chemotherapy. However, HBO did not increase the neovascularity, and they correlated the lack of chemotherapeutic potentiation to this. In a mammary tumor model, Moen et al. [48] found that the uptake of chemotherapy is increased for the duration of, and immediately after, HBO treatment. Based on this study, potentiation of chemotherapy can probably not occur unless the chemotherapeutic agent is administered during or immediately after the HBO session, when the pO_2_ is elevated. Another study by Moen et al. [24], on the same mammary tumor model, found altered genetic expression after HBO indicating a change to less tumorigenic metabolism, possibly influencing the chemotherapeutic response. Many have ascribed the enhanced chemotherapeutic effect after HBO to increased levels of ROS. Moen et al. [48], however, found no change in MDA levels after HBO, indicating that in this study ROS levels cannot be the main determinant of an increased chemotherapeutic effect. Microarray studies have made it possible to classify breast cancers at the molecular level [49, 50] and correlate their signatures with metastatic behavior and clinical outcome, and thereby making it easier to develop targeted therapy. Underlining the importance of breast cancer subtyping, it is important to comment on the differences between different tumor models: Moen et al. [48] found an increased uptake of the chemotherapeutic drug 5-FU into DMBA-induced tumors after HBO, while Jevne et al. [51] failed to find the same correlation in the 4T1 mammary tumor model.

The combination of HBO and chemotherapy has also been tried in other cancer types. Suzuki et al. [44] suggest that HBO therapy prolongs the biological residence time of carboplatin in glioma patients. However, there are still uncertainties concerning the mechanisms of action of HBO on the efficacy of carboplatin. The same group found that HBO enhanced transendothelial permeability in rat brains and HBO might therefore be favorable for the uptake and therefore also the effect of carboplatin [52]. Preliminary results from a small, clinical study, on nonsmall cell lung cancer, show promising results when combining hyperthermia and HBO with paclitaxel and carboplatin [45]. However, they emphasize that the study lacks proper controls, and thereby the additional value of HBO to the chemohyperthermia response cannot be made. Kawasoe et al. [21] found, both in vitro and in vivo, that HBO enhanced the chemotherapeutic effect of carboplatin in osteosarcomas. Furthermore, combining HBO and cisplatin significantly reduced tumor volume in a human ovarian cancer xenograft model [46].

It is, however, important to underline that Mayer et al. [53] list up five chemotherapeutic agents (doxorubicin, bleomycin, disulfiram, cisplatin, and mafenide acetate); all of which are strongly contradictory in combination with HBO due to potential potentiation of toxicity [54]. Of the reviewed papers, only Heys et al. [28] and Selvendiran et al. [46] have utilized the listed chemotherapeutics in combination with HBO.

Relating the knowledge on the different chemotherapeutics in relation to cancer subtypes will be important for further studies and for development of therapies and adjuvant therapies. In addition, proper randomized studies are necessary in order to be able to make any final conclusions regarding the effect of HBO in combination with chemotherapy.

### HBO and radiotherapy

Radiotherapy in combination with HBO has been used clinically in two different applications: (1) as a therapeutic agent for treating late radiation injury and (2) as a radiosensitizer, aiming to increase the effect of radiotherapy [53]. In this review, we focus only on the latter application of HBO.

Gray et al. [6] proved in the 1950s that the oxygen concentration influences the effect of radiotherapy and the influence of hypoxic modification in relation to radiotherapy has been extensively studied since then. In 2011, Overgaard published a meta-analysis reviewing the influence of hypoxic modification of radiotherapy in head and neck carcinoma [7]. Overall, Overgaard found that out of the various hypoxic modification techniques, HBO showed the most pronounced effect [7], and thus will improve the results of radiotherapy. Nevertheless, in a recent and extensive review by Bennett et al. [55], the authors have also reviewed the effect of radiotherapy in combination with HBO. They concluded that there is some evidence that HBO improves local tumor control and mortality in tumors of the head and neck; however, the outcomes seem to be related to the use of unusual fractionation schemes, and Bennett et al. [55] thereby conclude that the benefits of HBO should be interpreted with caution.

It has also been shown that adverse side effects like oxygen poisoning and severe tissue radiation injury is associated with the use of HBO in combination with radiotherapy [55]. However, it is important to emphasize the importance of timing of HBO exposure in relation to the radiation [53]. Kohshi et al. [56] found that to avoid hazardous side effects, irradiation should be administered immediately after and not concurrently to HBO treatment. It has been shown that euoxic conditions persist for some time after HBO exposure due to postponed oxygen saturation and washout kinetics [12, 13]. Thus, a change in protocols could possibly reduce or prevent serious side effects, and thereby justify the use of HBO in radiosensitization [53]. A conclusion regarding the use of HBO in combination with radiotherapy still remains unclear.

## HBO and cancer types

This review summarizes the work performed on HBO and cancer during the last 9 years (Table 1) and supports the previous findings [15, 16] since none of the studies reported a cancer-promoting effect of HBO. However, we have changed the focus to whether HBO might have an inhibitory effect on cancer growth. The variety of responses observed in cancers after HBO treatment supports what we know today, i.e., that no single treatment of any kind will be efficient in all types of cancers. However, could the treatment be efficient in some cancer types? And if so, why do we observe these differences?

**Table 1:** Studies on the effect of hyperbaric oxygen (HBO) and malignancy, both alone and in combination with conventional treatment, from 2001 to 2012

Study	Year	Type of study	Cancer type	HBO protocol	Additional therapy	HBO per se	Combo therapy	Metastasis	Angiogenesis
Breast cancer									
Stuhr et al. [47]	2004	In vivo	DMBA-induced mammary tumors in rats	0.2 MPa, 4 exp at 90 min, 11 days or 7 exp, 23 days	5-FU	↓	↓		
Granowitz et al. [18]	2005	In vitro	Mammary cells from normal epithelia, primary tumor, and metastatic tumor + human MCF7 cell line	0.24 MPa	Melphalan, gemcitabine, and paclitaxel	↓	↓		
Heys et al. [28]	2006	Clinical	Locally advanced breast carcinoma	0.24/0.2 MPa, 90 min daily (5/week) for 10 days	Cyclophosphamide, doxorubicin, and vincristine		↔		↔
Raa et al. [22]	2007	In vivo	DMBA-induced mammary tumors in rats	Hyperoxia (100 % O_2_) or 0.15 MPa, 4 exp at 90 min over 11 days	5-FU	↓	↓		↓
Haroon et al. [36]	2007	In vivo	Mouse mammary adenocarcinoma 4T1-GFP cell line in nu/nu mice	0.28 MPa for 45 min daily (5/week) up to 5 weeks				↓	
Moen et al. [24]	2009	In vivo	DMBA-induced mammary tumors in rats	0.2 MPa, 4 exp at 90 min, 11 days		↓			↓
Moen et al. [48]	2009	In vivo	DMBA-induced mammary tumors in rats	0.2 MPa, 4 exp at 90 min over 11 days or 1 exp at 90 min	5-FU		↓		
Jevne et al. [51]	2011	In vivo	Murine 4T1 mammary tumors in NOD/SCID mice	0.25 MPa, 3 exp at 90 min over 8 days	5-FU		↔		↓
Moen et al. [37]	2012	In vivo	Murine 4T1 mammary tumors in NOD/SCID mice	0.25 MPa, 90 min exp, 3 intermittent or 7 daily exp over 8 days		↓		(↔/↑)	↓/↔
Prostate cancer									
Chong et al. [29]	2004	In vivo	Human prostate (LNCaP) cells in immunodeficient mice	0.236 MPa, 20 exp at 90 min, 5/week for 4 weeks		↔ (↓)			↔
Tang et al. [31]	2009	In vivo	Human prostate PC-3 cells in immunodeficient mice	0.2 MPa, 20 exp at 90 min, 5/week for 4 weeks		↔			↔
Tang et al. [32]	2009	In vivo	Human prostate cancer LNCaP cells in immunodeficient mice	0.2 MPa, 20 exp at 90 min, 5/week for 4 weeks		↔			↔
Colorectal cancer									
Hjelde et al. [66]	2005	In vitro	Traditional cell carcinoma (AY-27), Human primary colonadenocarcinoma (WiDr) and human colonadenocarcinoma cell line (SW480)	0.1, 0.2, 0.3, and 0.4 MPa O_2_ for 30 min	Photodynamic therapy		↔		
Daruwalla et al. [38]	2006	In vivo	Dimethylhydrazine induced primary colon carcinoma cell line in mice	0.24 MPa, 90 min daily exp for 7, 13, 19, and 25 days		↓/↑		↔	(↔)
Daruwalla et al. [39]	2007	In vivo	Primary colon carcinoma cell line in mice	0.24 MPa, 5 times à 90 min over 9 days	SMA–pirarubicin	↔	↓	↓	
Gliomas									
Ogawa et al. [76]	2006	Clinical	Patients with high grade gliomas	0.28 MPa, 30-60 min	Radiotherapy and procarbazine, nimustine, and vincristine		↔/↓		
Stuhr et al. [23]	2007	In vivo	BT4C rat glioma xenografts in nude rats	100 % O_2_ or 0.2 MPa HBO, 3 exp at 90 min over 8 days		↓			↓
Kohshi et al. [75]	2007	Clinical	Patients with anaplastic astrocytoma and glioblastoma multiforme	0.25 MPa, 60 min	Radiotherapy (previous chemotherapy)		↔/↓		
Suzuki et al. [44]	2009	Clinical	Patients with recurrent malignant or brainstem gliomas	0.2 MPa, 60 min during i.v. adm. of carboplatin + 24 h after drug adm	Carboplatin		↓		
Other									
Chen et al. [20]	2007	In vitro	Human leukemia (Jurkat), multiple myeloma (NCl-H929), carcinoma (A549) and breast adenocarcinoma (MCF-7) cell lines	0.25 or 0.35 MPa oxygen or air for 2–12 h		↓/↔			
Ohgami et al. [43]	2010	In vitro	Molt-4 human leukemia cells	0.35 MPa, 90 min	Artemisinin	↓	↓		
Sun et al. [19]	2004	In vivo	Human oral cancer cell line in mice	0.25 MPa, 20 exp. at 90 min		↔			
Shi et al. [27]	2005	In vivo	Head and neck squamous cell carcinoma (Sq20B and Detroit 562) in mice	0.24 MPa, 90 min 5 times a week for 2–4 weeks	Radiotherapy (single dose)	↔	↔		↔
Schönmeyr et al. [30]	2008	In vitro and in vivo	Murine squamous cell carcinoma (SCC-VII) cell line in vitro and in mice	0.21 MPa 8 daily exp à 90 min		↔			↔
Ohguri et al. [45]	2009	Clinical	Patients with non-small-cell lung cancer (NSCLC)	0.2 MP, 60–90 min, after chemo and HT	Paclitaxel and carboplatin		↔/↓		
Kawasoe et al. [21]	2009	In vitro and in vivo.	Mouse osteosarcoma (LM8) cell line in vitro and implanted in mice	0.25 MPa for 90 min	Carboplatin	↓	↓	↓	
Selvendiran et al. [46]	2010	In vivo	Human ovarian cancer xenograft	0.2 MPa, 90 min daily for up to 21 days	Cisplatin	↓	↓		
Peng et al. [25]	2010	In vitro	Nasopharyngeal carcinoma CNE2Z cells	0.2 MPa, 85 % O_2_, exp at 90 min (4 h interval)	5-FU	↓/↔	↓		

*Left–right arrow* no effect, *down arrow* inhibition/reduction, *up arrow* potentiation (if two symbols are given, the effect is mixed), *Combo* combination, *exp* exposure, *adm* administration, *HT* hyperthermia

### HBO and breast cancer

Breast cancer is the most frequently occurring cancer in women and comprises 22.8 % of cancer incidence in females worldwide [57]. Granowitz et al. [18] showed that HBO treatment alone had a strong antiproliferative effect on different mammary cancer cells in vitro. They suggested that HBO could be an effective therapy for breast cancer. This is supported by six different animal studies performed during the last 9 years, using clinically relevant HBO protocols. These revealed a significant inhibitory effect of HBO as a stand-alone treatment on mammary tumor growth in vivo [22, 24, 37, 47, 48, 51] (Table 1). Feldmeier et al. [15] and Daruwalla et al. [16] reviewed three older studies on mammary tumors and HBO, all in the same C3H mouse model, where none of them found effects on tumor growth [58–60]. However, they did not consider an extensive study from 1964 in their reviews, where Kluft et al. [61] reported that HBO retarded growth of a transplanted mammary carcinoma (TM 8013) growing in C 57 black mice.

As the main focus in the older studies was to confirm or reject HBO as cancer promoter, most studies focused only on cancer growth and metastasis. Nevertheless, several recent studies, showing cancer inhibitory effects, have gone into more detail. As previously mentioned, HBO has been shown to induce an antiangiogenic effect in two mammary tumor models [22, 48, 51]. Furthermore, an increase in cell death and reduced cell proliferation, together with a significant change in histology, has also been shown after HBO treatment in DMBA-induced mammary tumors in vivo [22, 24]. In relation to metastasis, it has been shown that HBO induced MET in DMBA-induced mammary tumors, leading to a less aggressive tumor type [24]. In a 4T1 mammary tumor model, Haroon et al. [36] found that HBO restricts the growth of large tumor cell colonies. Moen et al. [37] found lung metastasis in the same tumor model after HBO, thus HBO here did not hinder metastasis. However, they lack comparable endpoint controls and therefore a conclusion as to whether there would be less colonies could not be drawn.

Despite a significant number of animal studies, no clinical trials on HBO and breast cancer per se have been performed and only one small clinical study on combined treatment is available. With this background, we conclude that the effect of HBO should be further explored in breast cancer subtypes, especially focusing on the possible effect of HBO as an adjuvant tumor therapy.

### HBO and head and neck cancer

The National Cancer Institute defines head and neck cancer as a neoplasm that arises in the nasal cavity, sinuses, lips, mouth, salivary glands, throat, or larynx [62]. Only one study has been performed during recent years, where HBO has been studied in combination with radiotherapy in experimental head and neck carcinoma in mice [27] (Table 1). They found that even though HBO did reduce the hypoxic state of the tumors, it did not have any effect on tumor growth, neither alone nor in combination with radiotherapy [27]. Furthermore, they did not find evidence of enhanced angiogenesis in the tumors after HBO treatment, neither when staining for CD31 nor measuring VEGF expression, supporting the notion that HBO does not induce angiogenesis in tumors.

As previously stated, Bennett et al. [55] reviewed the effect of combining HBO with radiotherapy. Even though studies have shown beneficial results on local tumor control, mortality, and local tumor recurrence, the protocols of the reviewed literature made them conclude that they could not justify the routine use of HBO in combination with radiation [55]. However, as discussed in “HBO and radiotherapy,” the conclusion within the field of HBO and radiosensitization has not yet reached a consensus.

### HBO and colorectal cancer

Colorectal cancer is a disease originating from the epithelial cells lining the colon or rectum of the gastrointestinal tract [63]. Most colorectal cancers occur due to lifestyle and increasing age with only a minority of cases associated with underlying genetic disorders [64]. Even though surgery can be curative if the disease is caught early, additional treatment of advanced colorectal cancer is commonly in use [63].

Several studies have examined the effect of HBO concomitant with other therapies in colorectal cancer. In an older clinical study, Dische and Senanayake [65] demonstrated positive results when combining HBO and radiotherapy on patients with carcinoma in the colon and the rectum. Hjelde et al. [66] studied the effect of hyperoxia in combination with photodynamic therapy on three different colon carcinomas in vitro (Table 1). They concluded that hyperoxia did not increase the occurrence of cell death after photodynamic therapy. However, older experimental and clinical studies have demonstrated that HBO improves the effect of photodynamic therapy [67–71]. Thus, the lack of response in the study by Hjelde et al. [66] might be ascribed to lack of hypoxic cells in the in vitro experimental setup. Additionally, two papers by Daruwalla et al. [38, 39] examine the effect of HBO in two different in vivo colon tumor models (Table 1). In the first paper, the effect of HBO per se was studied [38]. Here, they concluded firstly that HBO did not have any tumor stimulatory effect and does not promote formation of distal metastases, and secondly that HBO therefore can safely be used in combination with other therapies. Furthermore, they performed experiments on an in vivo model of primary colon carcinoma with HBO both alone and in combination with styrene maleic acid (SMA)–pirarubicin [39]. Again, they concluded that HBO alone gave no effects. However, HBO in combination with SMA–pirarubicin gave a reduction both in liver metastases and tumor growth, in addition to inducing increased levels of necrosis. Thus, HBO as a stand-alone treatment seems to have no effect on colorectal cancer, but as a treatment adjuvant, HBO seems to be an interesting alternative and its potential use should be explored further.

### HBO and gliomas

Gliomas are tumors originating in the glial cells in the brain or the spine. Patients with high-grade gliomas generally have poor prognosis [72], and the illness is rarely curable. Designing therapy is challenging due to the neoplasm’s infiltrative nature, resistance to apoptosis, and recurrence and resistance to therapy [73]. In 2011, Beppu et al. [74] reviewed the effect of HBO on gliomas. However, the review only exists in Japanese, and thus is not commented on.

In 2007, Stuhr et al. [23] published an experimental study, examining the effect of HBO on the growth and development of rat glioma xenografts per se (Table 1). They found that increased levels of pO_2_, using both normobaric and moderate HBO, significantly reduced tumor growth, possibly by increasing cell death and reducing the vascular density. This might indicate that HBO alone has a favorable effect on gliomas. However, it is important to underline that the experimental tumors were implanted in the neck and not in the brain, and this may well have influenced the outcome of the experiments.

Further, only three other papers in the period 2004–2012 have been published utilizing HBO on gliomas (Table 1). They are all preliminary clinical studies, investigating HBO in combination with radiotherapy and chemotherapy [44, 75, 76]. Kohshi et al. [75] and Ogawa et al. [76] both conclude that there is a possible advantage to combining HBO with radiotherapy, but they also underline the need for further investigation within this field. Special caution should be taken when interpreting the results from the study by Koshi et al. [75], as anaplastic astrocytomas are included in the trial and compared with the patients with glioblastoma mulitforme.

In a study of HBO and chemotherapy, Suzuki et al. [44] suggest that HBO therapy prolongs the biological residence time of carboplatin. However, the mechanisms of action of HBO on the clinical efficacy of carboplatin are still unknown. Some evidence implies that HBO as an adjuvant to traditional therapy in gliomas should be investigated further, and this could lead to an improvement of current therapy regimens.

### HBO and leukemia

Leukemia is cancer of the blood or bone marrow characterized by an abnormal increase of immature white blood cells [77]. Two recent in vitro experiments have shown promising results when treating leukemia cells with HBO [20, 43] (Table 1). In addition, Tonomura and Granowitz [78], in an editorial in 2007, have commented on the effect of HBO on leukemia. They concluded that since HBO promotes apoptosis in leukemia cells, it should be further exploited as a novel treatment for leukemia. It is, however, important to emphasize that this is based on experiments performed in cell culture, and thus needs further validation from in vivo models to exclude the possibility that this is just an in vitro phenomenon. In two older experiments, studies were performed on HBO using animal leukemia model systems [79, 80]. In neither of the in vivo experiments were differences observed in growth rate or metastasis after HBO treatment. However, the limited number of studies might therefore call for further investigation with regard to the use of HBO in leukemia.

### HBO and prostate cancer

Cancer of the prostate gland is the second most frequent type of cancer in men worldwide, accounting for 13.6 % of all cases [57]. Treatment of prostate cancer depends on the grade of the disease. As most prostate cancers are slow growing, some cancers are not treated at all. However, aggressive cancers are normally treated using surgery, in addition to chemotherapy, hormonal therapy, immunotherapy, and/or radiation.

Three animal studies have been published recently on HBO as stand-alone treatment of prostate cancer (Table 1). Neither Chong et al. [29] nor Tang et al. [81, 32] found any change in in vivo tumor growth after HBO treatment. None of the pathological characteristics, such as microvessel density, differentiation status, proliferation, or apoptosis, were changed. In addition, Kalns et al. [82, 83] published two papers in the late 1990s where they showed that HBO can decrease the rate of growth and increase the sensitivity to the anticancer agents taxol and doxorubicin in in vitro experiments, by accumulating prostate cancer cells in the chemosensitive portion of the cell cycle. Further studies on in vivo prostate cancer models and the effect of HBO as an adjuvant to chemotherapy are evidently necessary before any definite conclusions can be made.

### HBO and cervical cancer and bladder cancer

Cervical cancer of the female reproductive system represents 8.8 % of cancer incidence in women and bladder cancer 3.0 % in both sexes [57]. Based on ten clinical studies, Daruwalla et al. [16] stated that HBO treatment of patients with cervical and bladder cancer did not offer any improved benefit or improved outcome. The older clinical trials, combining HBO and radiotherapy, generally showed no change in cancer growth or survival. This is presumably the reason why no new studies have been performed on the effect of HBO on these cancer types. Thus, neither cervical cancer nor bladder cancer seems to be good candidates for demonstration of an improved effect of traditional therapy in combination with HBO.

## Comments and future work

The consensus today is that research performed hitherto has failed to demonstrate that HBO has a cancer-promoting effect or that it enhances recurrence. Nevertheless, both recent and older research studies have shown that HBO can be inhibitory and reduce cancer growth in some cancer types, like breast cancer. On the other hand, cervical and bladder cancers appear to be nonresponders to HBO. In vitro studies have confirmed that there are discrepancies in growth fractions between different cancer cell lines following exposure to hyperoxia [10]. Thus, this supports the need for performing randomized studies on HBO as a stand-alone treatment or in combination with other therapies for certain cancer types or subtypes.

The observed variety in response to HBO found during the last decades can be ascribed to both differences in types of cancers but also to the large variety in HBO treatment protocols. Thus, differences in response to oxygen between different cancer types should not lead to an exclusion of HBO as a form of cancer treatment or as a cancer treatment adjuvant for selected types of cancers. Further research on HBO and its effect on certain types of cancer and studies on the underlying mechanisms involved are therefore needed.

To clarify if tumor hypoxia is as important for cancer progression as indicated in the literature, HBO can be used as an important research tool. Concomitant studies of hyperoxia (“the flip of the coin”) and hypoxia might be valuable and can give us additional and important information on how oxygen influences cancer growth and metastasis. We therefore strongly believe that we need to expand our understanding of what happens during oxygenation of cancer tissue and we need to examine in depth the effect of hyperoxia on different cancer types and subtypes.
